# Split Crest—Is It Necessary to Fill the Gap?—A Controlled Trial

**DOI:** 10.3390/jfb16120467

**Published:** 2025-12-18

**Authors:** Vladimir Biocanin, Zoran Tambur, Djordje Pejanovic, Marija Biocanin, Mihailo Ostojic, Marija Lalovic, Svetislav Zaric

**Affiliations:** 1 Faculty of Stomatology in Pancevo, University of Business Academy in Novi Sad, 179 Zarka Zrenjanina, 26000 Pancevo, Serbiadjordje.pejanovic@stomatoloski.rs (D.P.);; 2 Private Practice, 11060 Belgrade, Serbia; 3 Guy’s Hospital, King’s College London, London SE1 9RT, UK

**Keywords:** split crest, dentin graft, alveolar ridge splitting, bone expansion, bone density, implant stability

## Abstract

Introduction: The split crest (SC) is a technique for horizontal ridge augmentation that enables simultaneous implant placement. While the use of bone grafts within the osteotomy gap is well-documented, the efficacy of dentin as a graft material in SC procedures has not been thoroughly evaluated. Objective: This study aimed to assess whether the addition of bone graft or dentin to the osteotomy gap during the SC procedure improves bone width, density, and implant stability compared to SC without grafting. Materials and Methods: A partially randomized prospective study was conducted on 24 implants divided into three groups: SC only, SC and bone graft, and SC and dentin graft. Clinical and radiographic evaluations, including CBCT-based bone measurements and implant stability (ISQ) values, were performed preoperatively, immediately postoperatively, and three months after surgery. All patients were followed for one year. Results: Significant increases in bone width were recorded in all groups, with gains exceeding 2 mm in the SC and SC and bone graft groups. The SC and dentin group showed the least bone gain and the greatest horizontal bone resorption (*p* < 0.05). Conclusions: While bone grafting may offer modest advantages in bone preservation, the use of dentin grafting may not demonstrate significant benefits.

## 1. Introduction

The split crest (SC) is a surgical technique often used in dental implantology to enhance the width of the alveolar ridge before implant placements. This technique is especially useful when the ridge has primarily undergone horizontal resorption but still retains sufficient height for implant placement [1,2]. SC is a reliable technique with great advantages over guided bone regeneration, which is still considered the gold standard. Namely, with SC, installation of dental implants can be simultaneous, decreasing the number of surgical procedures and the time of the treatment and also the need for bone augmentation and possible complications related to it, such as donor site morbidity, nerve injury, unpredictable bone resorption, wound dehiscence, and infection of the grafted material [3,4].

The split crest technique is a technically demanding procedure suitable only for carefully selected cases and experienced surgeons that reduces the need for bone grafting. This technique is indicated in situations when the thickness of the alveolar ridge is 3.5 mm or greater, with sufficient height [5]. Local contraindications for SC include absence of cancellous bone, undercuts of the alveolar ridge and short edentulous spaces [6].

According to previous studies, the average gain in the horizontal dimension with SC was 2.97–3.8 mm [7,8]. When performed in carefully selected cases, SC has a high success rate, up to 98.8% in the five years post-operation, and stable long-term results [9]. The survival rate of immediately placed dental implants with SC in human studies ranged from 91.7% to 100%, with an average observation period ranging from 12 months to 10 years [9,10].

Recent studies showed different results regarding application of the bone substitutes into the gap between cortical plates. Ella et al. [11] compared SC with and without bone augmentation material and they found significantly less horizontal bone resorption postoperatively in the grafted cases with biphasic calcium phosphate (hydroxyapatite 60% and beta-tricalcium phosphate 40%), whilst Tang et al. [12] found no difference between SC in combination with deproteinized bovine bone mineral which was covered with a resorbable collagen membrane, when compared to SC without it.

Dentin grafts are derived from the patient’s own extracted teeth. Their use eliminates donor site morbidity compared to autografts and simultaneously reduces costs relative to allografts and xenografts. The use of dentin as a grafting material shows many advantages over other bone substitutes, such as complete biocompatibility, a lower risk of rejection, and a reduced chance of infection. Furthermore, dentin grafts are rich in growth factors, which promote bone regeneration. A systematic review of Mahendra DA et al. [13] showed that dentin grafts for alveolar augmentation of post-extraction sockets or other alveolar bone defects were effective in volume maintenance, indicating promising results via histomorphometry and radiographic analysis. The efficiency of using an autogenous dentin graft showed similar results in comparison to using a deproteinized bovine bone in relation to buccolingual socket reduction following immediate implantation [14]. However, no study has yet evaluated the results of applying autologous dentin chips into the gap between cortical plates using SC and simultaneous implant installation.

The null hypothesis is that there is no difference among implants placed with SC, SC and dentin, and SC and an alloplastic bone substitute in terms of bone width, bone density, and implant stability.

The aim of this study was to assess how the addition of autologous dentin in conjunction with SC and simultaneous implant placement compares to SC only and SC and alloplastic bone graft in relation to bone width, bone density, and implant stability.

## 2. Material and Methods

This study adhered to the AGREE guidelines [15]. This study was approved by the Ethical Committee of the Faculty of Stomatology in Pancevo, Serbia, No 999/2-2024. Participants gave informed consent before taking part and the study was conducted in accordance with the Declaration of Helsinki, as revised in 2013. The study was a prospective single-center controlled trial and the trial is registered on ClinicalTrials.gov under the number NCT07272694.

The inclusion criteria for the study were (a) a minimal horizontal bone thickness of 4 mm; (b) a minimal vertical bone height of 10 mm; (c) no undercuts in the alveolar bone profile; and (d) at least one hopeless tooth indicated for extraction (in the SC and dentin group). The exclusion criteria were as follows: (a) presence of any systemic disease; (b) alcohol or drug abuse; (c) heavy smoking (more than 10 cigarettes per day); (d) presence of oral tumors and/or other mucosal lesions; (e) presence of infection and/or tooth remnants in the surgical site; (f) poor oral hygiene; (g) presence of parafunctional habits (clenching and/or bruxism); (h) use of any medications; (i) pregnancy or breastfeeding.

This preliminary study included twelve patients who received twenty-four dental implants inserted in both jaws. The sample consisted of five females and seven males, with four patients allocated to each group (Table 1). Eight tapered implants 3.5 mm and 4 mm in diameter (TSIII^®^ dental implants, Osstem, Seoul, Republic of Korea) were placed in each group, with two implants per patient. The participants were allocated to one of the three groups: Group I—SC and simultaneous implantation without grafting (SC); Group II—SC and simultaneous implantation with bone grafting (SC and bone graft); Group III—SC and simultaneous implantation with dentin grafting (SC and dentin). The patients were randomly allocated to the SC or SC and bone graft group, but with the SC and dentin group we could not select patients randomly. In that group, we included only patients who had teeth indicated for extraction and we then milled the extracted teeth using a dentin grinder. All patients were ≥50 years old. Implant placement locations varied (anterior/posterior; maxilla/mandible), but most implants were placed in the posterior mandible (Table 1).

### 2.1. Preoperative Procedure

**Figure 1:**
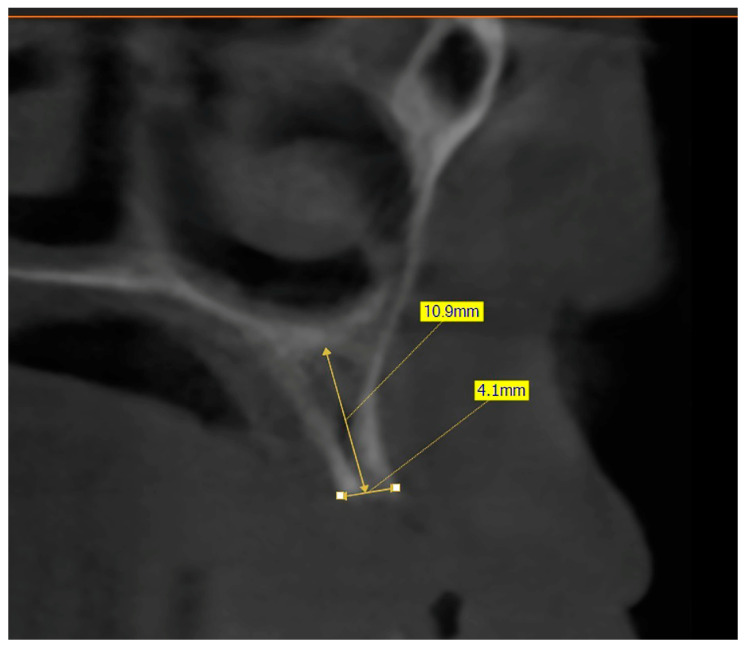
Bone measurement on a cross-sectional image—CBCT (Green X™ 12^®^, Vatech, Prague, Czechia).

**Figure 2:**
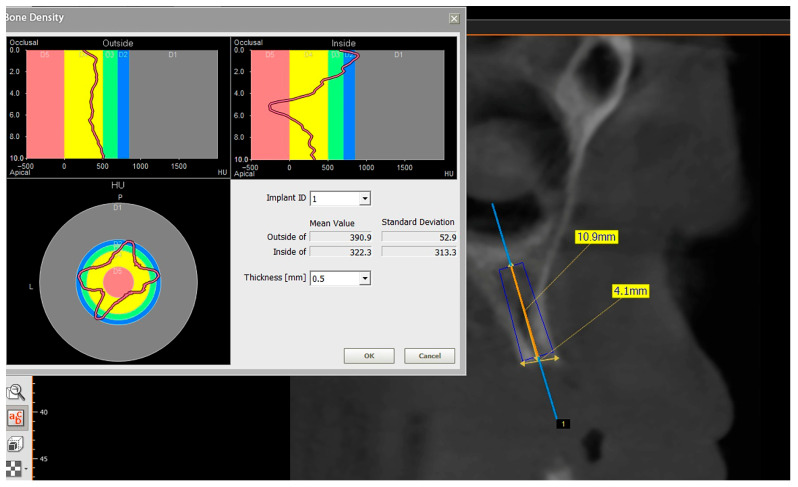
Bone density around a virtually placed dental implant—CBCT (Green X™ 12^®^, Vatech, Prague, Czechia).

The preoperative plan included a detailed history, clinical examination, and analysis of 3D CBCT images. Detailed measurements of bone height and width at the sites of the future implants (at the level of planned position of implant platform), as well as bone density, were performed on cross-sectional CBCT images (Figure 1 and Figure 2) by an investigator who was not involved in the surgical procedure. Prior to the surgery, an oral rinse of chlorhexidine digluconate solution 0.2% (Hibideks DAP^®^, Galenika, Beograd, Serbia) was administrated to all the patients for 1 min.

### 2.2. Split Crest Procedure

**Figure 3:**
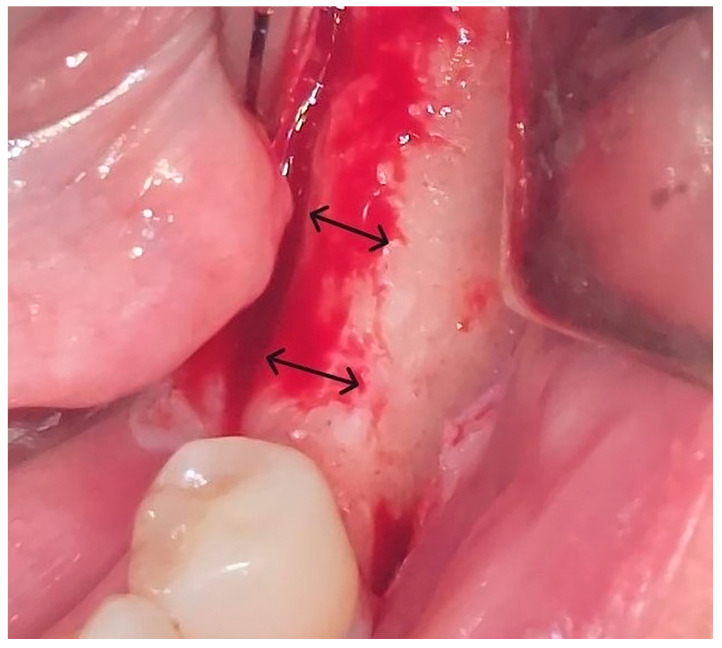
Flattening of the top of the alveolar crest to the level of 4 mm.

**Figure 4:**
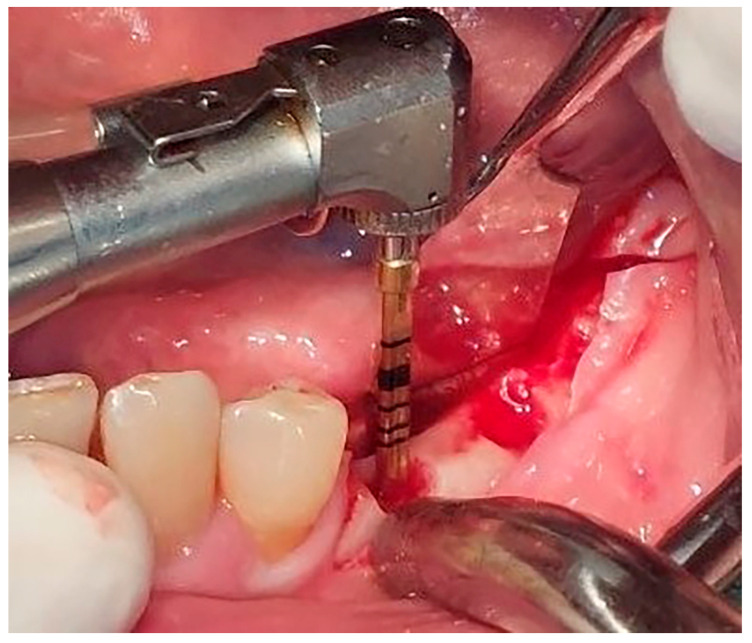
Preparation of the implant bed.

**Figure 5:**
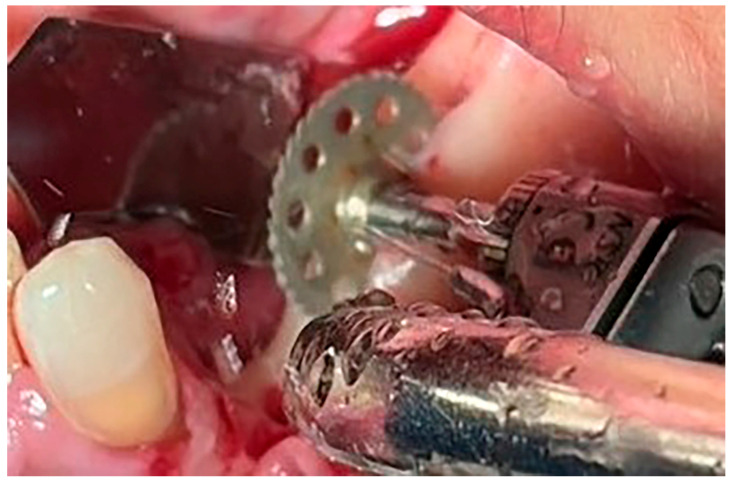
Midcrestal osteotomy with a saw.

**Figure 6:**
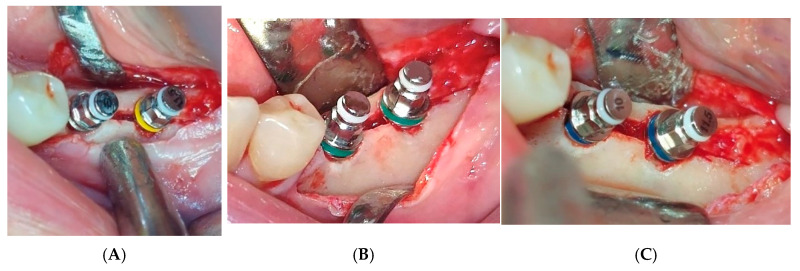
(**A**–**C**) Bone expansion with a gradually increasing bone expander diameter.

Local anesthesia using 4% articaine with adrenaline 1:100,000 (Septanest^®^, Septodont, Saint-Maur-des-Fossés, France) was administered. After the midcrestal longitudinal incision, a full thickness mucoperiosteal flap without a vertical releasing incision was elevated and the top of the alveolar ridge was flattened to the width of 4 mm (Figure 3). Preparation of the implant bed was performed with only one twist drill from the set (Esset kit^®^, Osstem, Seoul, Republic of Korea) which corresponded to the implant length (Figure 4). Following the preparation of the implant bed with the twist drill, a midcrestal longitudinal osteotomy was performed with a special saw with the speed of 1200 rpm (Figure 5). A specially designed set of expanders (Esset kit^®^, Osstem, Seoul, Republic of Korea) was used gradually in order to separate the buccal plate from the lingual cortical plate, according to their elasticity (Figure 6A–C).

**Figure 7:**
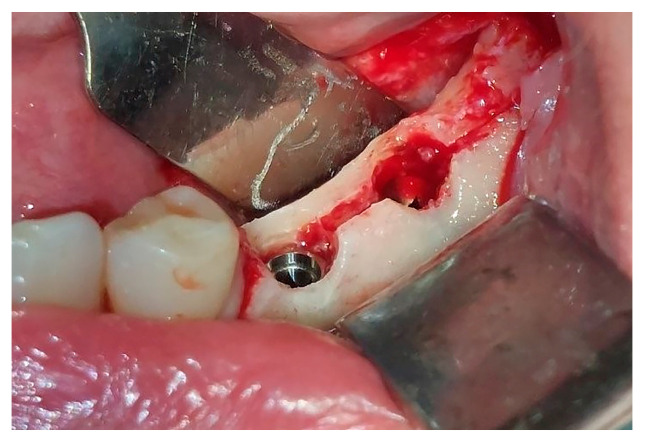
Subcrestal position of inserted implants.

**Figure 8:**
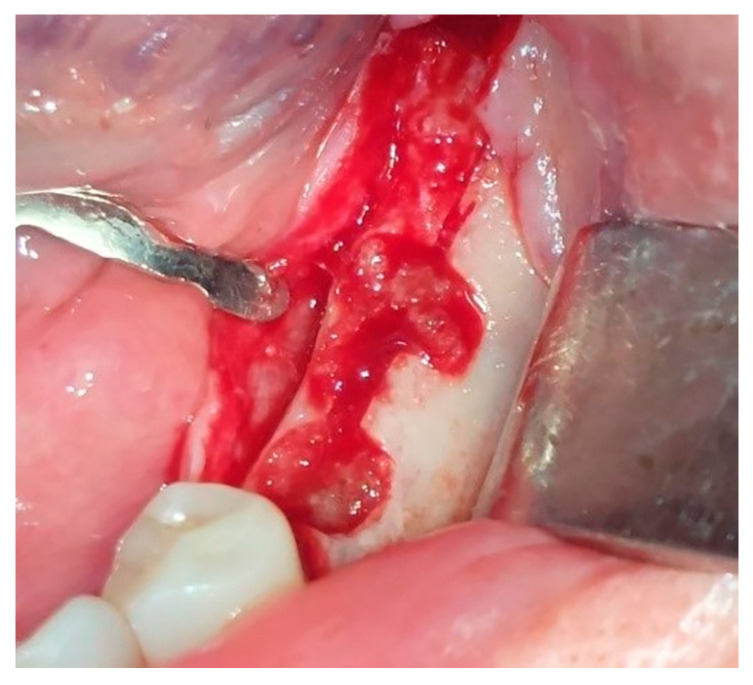
Bone graft placed between buccal split from lingual bone lamella after implant placement.

The maximum insertion torque of the expanders was 50 Ncm at a speed of 35 rpm. Implants were inserted with the maximal torque of 40 Ncm and positioned 1.5 mm subcrestally (Figure 7). The cut-off value of ISQ (Implant Stability Quotient) used to define adequate stability and minimal micro-mobility of inserted dental implants was 65. Bioactive glass (GlassBone^®^, Noraker, Villeurbanne, France) was used as an alloplastic material in Group II and was placed in the gap created by the expanders between the buccal and oral bone plates (Figure 8).

### 2.3. Dentin Chip Preparation

**Figure 9:**
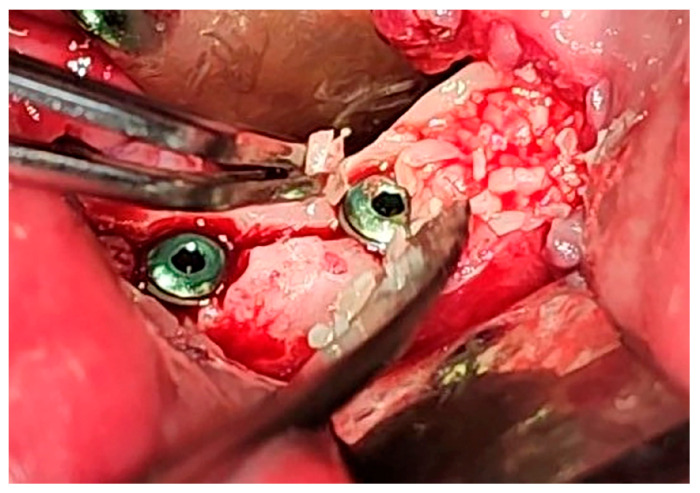
Dentin particles placed between buccal split from lingual bone lamella.

In Group III, the tooth indicated for extraction was extracted and milled in a dentin grinder (The Smart Dentin Grinder^®^, Tenafly, NJ, USA) and the gap between the cortical plates was filled with dentin (Figure 9). Periodontitis was the most common reason for extraction. After the extraction, the surface of the extracted tooth was cleaned of any remnants of soft tissue, composites, endodontic material, or decay using tungsten burs. There was no need to remove the enamel. Then, we placed the tooth into the chamber, set GRIND to a grinding duration of 3 s, set SORT to 10 s, and started the process. After the grinding was complete, the top drawer contained particles between 300 and 1200 µm and the bottom drawer contained particles smaller than 300 µm. We mixed the dentin particles together and proceeded with disinfection. The graft was completely soaked with Dentin Cleanser and left for 5 min. The cleanser removed all organic substrate and left the graft bacteria-free. Sterile gauze was used to dehydrate the cleanser. Then we applied Dentin Wash (phosphate-buffered saline) to completely cover the graft, immediately dehydrated it with fresh sterile gauze, and repeated this step twice. After that, the dentin graft was ready for use and we inserted it into the gap created with expanders between the buccal and oral bone lamellae.

**Figure 10:**
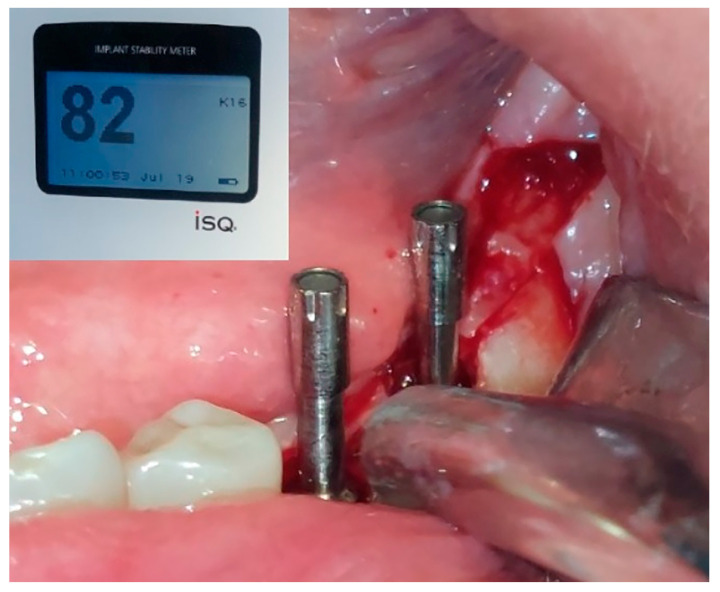
Measurement of ISQ values with smart peg and Osstell Mentor.

Initial implant stability was measured using the Osstell Mentor^®^ (Osstell AB, Gothenburg, Sweden) device and expressed in ISQ (Implant Stability Quotient) values (Figure 10). Implants were covered with cover screws and the wounds were sutured using PTFE sutures (USP 4-0) (Cytoplast^TM®^, Braintree, MA, USA). A CBCT scan was performed immediately after the operation to measure the width of the alveolar ridge at the level of the implant platform, as well as the bone density around the implants in Hounsfield units. Postsurgical analgesic treatment was administered with 400 mg ibuprofen every 6 h for 3 days; 1 g amoxicillin + clavulanic acid (Amoksiklav^®^, Sandoz, Kundl, Austria) was prescribed twice a day for 5 days. Suture removal was performed 7 days following the surgery. Healing was uneventful in all cases, with no complications observed at the surgical sites.

### 2.4. Postoperative Evaluation

Patients were re-assessed three months after the procedure. Following a clinical examination, CBCT (Green X™ 12^®^, Vatech, Prague, Czechia) scans were performed and measurements repeated at the same sites by the same investigator who was not involved in the operation. The post-implant bone width was measured at the level of the implant platform on CBCT cross-sectional images from the most lateral point of the bone on the vestibular and oral sides. Measurements were performed using a linear ruler, always at the same high magnification (one cross-sectional image per window). All CBCT scans were performed using the same CBCT unit (Green X™ 12^®^, Vatech, Prague, Czechia) and the same software. One independent examiner analyzed all CBCT images (before, immediately after, and 3 months postoperatively). Voxel size was the same—0.1 mm for all the samples. Local anesthesia was administered, the cover screw was removed, and the smart peg was placed into the dental implant to measure the ISQ. Subsequently, healing abutments were placed. All implants were loaded following the conventional loading protocol, using metal–ceramic screw-retained or cemented bridges or implant supported dentures. Patients were followed up one year postoperatively.

### 2.5. Statistics

Statistical analysis was performed using statistical software SPSS v.20.0 (IBM Corporation, Armonk, NY, USA). The Kruskal–Wallis test was used in the study given the small sample size. The level of significance was 0.05. The study included 24 implants, collected over a two-year period to satisfy strict inclusion criteria. Further enlargement of the sample size would have rendered the study obsolete due to rapid advancements in the field. There was no similar study using SC and dentin grafting, so we could not calculate sample size. We previously conducted a preliminary (pilot) study on patients who were not included in the final study (e.g., heavy smokers and patients with systemic diseases), which demonstrated that significant differences could be detected even with a small sample size (eight implants per group).

## 3. Results

There were no fractures of the buccal or lingual bone lamellae in any of the surgical sites.

**Figure 11:**
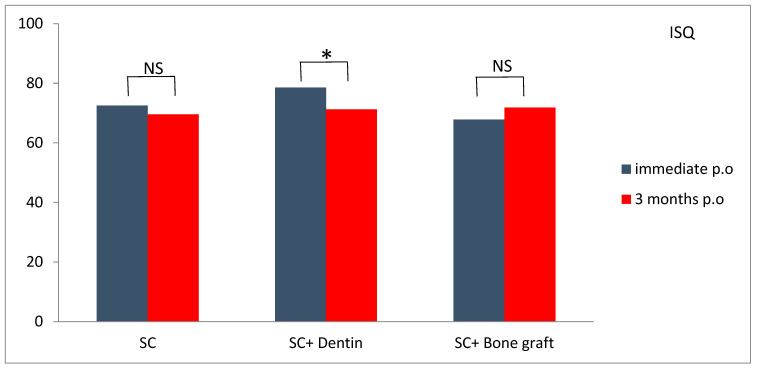
Comparison of mean ISQ values measured immediately postoperatively and at three months in each group (NS—nonsignificant; * significant *p* < 0.05).

All inserted implants showed high initial stability, with ISQ values over 65. Likewise, 3 months postoperatively, all implants showed ISQ values ≥ 65. Implants in the SC and dentin group showed a statistically significant decrease in ISQ values 3 months postoperatively compared to initial stability (Figure 11). Although ISQ values in the SC and dentin group significantly decreased (*p* < 0.05) compared to the SC and SC and bone graft groups (Table 2), this difference was not clinically relevant.

**Figure 12:**
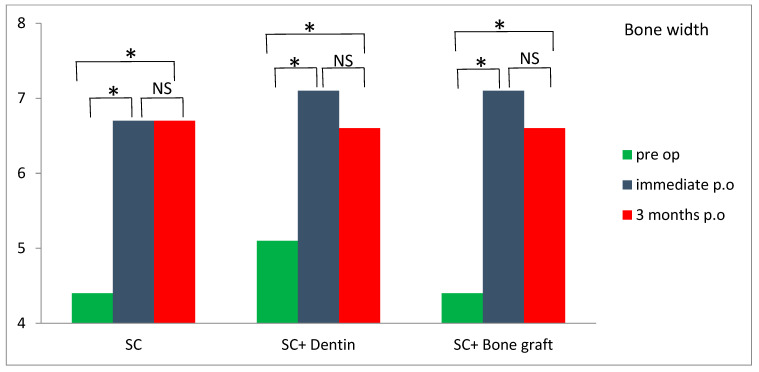
Comparison of bone width within the test groups (NS—nonsignificant; * significant *p* < 0.05).

An increase in alveolar bone width was obtained in all three groups three months postoperatively compared with preoperative values (Table 2). A bone width gain of more than 2 mm was observed in both the SC and SC and bone graft groups. Although the difference was not statistically significant (*p* > 0.05), greater bone width values were observed in the SC and SC and bone graft groups compared to the SC and dentin group three months postoperatively, relative to preoperative values. Horizontal bone resorption (difference between postoperative and 3 months review) was significantly higher in the SC and dentin group compared with the SC group (*p* < 0.05) (Table 2). A significant increase in bone width was observed in all of the tested groups three months postoperatively compared to preoperative values (Figure 12).

**Figure 13:**
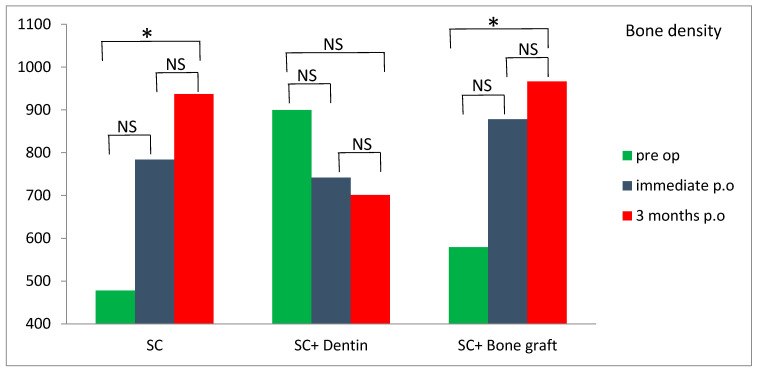
Comparison of bone density within the tested groups (NS—nonsignificant; * significant *p* < 0.05).

Significantly higher values of bone density were measured 3 months postoperatively compared to preoperative values in the SC and SC and bone graft group (*p* < 0.05) (Figure 13). However, the SC and dentin group showed a slight decrease in bone density at 3 months following the surgery (Figure 13). Survival rate of dental implants was 100% after one year follow-up.

## 4. Discussion

Our study found high ISQ values in all three test groups, both immediately after the implant placement and three months later (with most implants showing ISQ values higher than 70). This implies that all the implants could be immediately loaded [16] when implanted simultaneously with the SC technique. High values of ISQ immediately following SC and implantation could be attributed to elasticity of buccal and lingual bone lamella and slight compression to dental implants between them. Three months following the operation, ISQ values were also high, and this could be explained by excellent osseointegration of dental implants. Although, after three months, the ISQ values were significantly lower in the SC and dentin group than in other groups, it was clinically irrelevant, considering that all ISQ values were still over 65. The null hypothesis was rejected.

The average horizontal bone gain at the level of the implant platform in the SC, SC and bone graft, and SC and dentin groups was 2.46, 2.18 and 1.52 mm, respectively. This is lower than the results of the study by Waechter et al. [8] who found an increase in bone thickness of 3.8 mm. De Souza et al. [7], in their study using SC with simultaneous implantation and filling the gap with bovine bone, found an increase in bone thickness of 2.97 mm.

Dimensional changes in bone thickness are expected after SC. In our study, the mean reduction in bone thickness 3 months postoperatively, compared to immediately postoperatively, was 0.06, 0.49, and 0.83 mm in the SC, SC and bone graft, and SC and dentin groups, respectively. There was no significant difference in bone loss between the SC and SC and bone graft groups. This is in-line with the study by Tang et al. [12], who found no difference between the results of SC with GBR compared to SC without GBR. Likewise, a study in mini pigs demonstrated no differences regarding bone healing in the gap region between split bone plates comparing sites with and without filling of the bone gap with calcium phosphate substitute, as well as comparing sites with and without the application of a barrier membrane after a follow-up of 6 weeks [17]. On the contrary, Ella et al. [11] found significantly less horizontal bone resorption with SC and biphasic calcium phosphate with a barrier membrane compared to SC without bone augmentation. The possible for reason why we did not find significantly better results with SC and bone graft compared to SC alone may be, firstly, that we only used a bone substitute without a barrier membrane and secondly, that the buccal bone plate was ≥1.5 mm, which is resistant to resorption [18]. In one dental implant from the SC and dentin group, we observed vertical and horizontal buccal bone resorption 2 mm apical to the implant platform. The probable reason for this the initial thickness of the buccal bone plate being less than 1.5 mm, combined with the elevation of the mucoperiosteal flap. In the study by Bassetti et al. [6], the most bone loss occurred in the control group (split crest and simultaneous implantation without GBR) during the unloaded healing period after implant surgery (0.94 ± 0.78 mm). In our study, the average bone loss during the unloaded healing period after implant surgery in the SC and bone graft group was 0.49 ± 0.75 mm.

A significant reduction in bone thickness was observed in the SC and dentin group compared to the SC group three months postoperatively. This finding may be attributed to relatively high osteoclast activation and resorption of the dentin and bone surface. This is in accordance with the experimental study by Shilling et al. [19], who seeded osteoclast cell culture on the surface of dentin and, after 4 weeks, found 75.5% surface resorption. In addition, Olchowy et al., 2024 [20] showed that there was a lower increase in the migration of human fetal osteoblastic cells when they were cultured with dentin material compared to allograft (44.4% vs. 59.2%) on day 3.

There is still no study that has measured bone density around dental implants placed simultaneously with SC. Although a CBCT scan is not a precise method for measuring bone density, it may serve as a non-invasive proxy measurement. In our study, bone density was measured on CBCT before the surgery, immediately after the surgery and 3 months following the surgery in the region of implant placement. Bone expansion with SC increased bone density in all three groups immediately after the surgery, due to lateral condensation of spongious bone with tapered designed bone expanders. Bone density significantly increased 3 months postoperatively in the SC and SC and bone graft groups compared to the SC and dentin group, where bone density slightly decreased. This could be explained by potential better bone healing and osseointegration in the SC and SC and bone graft groups compared to the SC and dentin group.

It is important to note the clinical difference between survival and success rates. Only the success-rate data can capture the complications associated with implant therapy. Many different success criteria have been suggested [21,22,23]. A limitation of our study was that we considered only the survival rate of dental implants after one year and not the success rate, which is a more sensitive measure. According to the literature, survival rate of dental implants using a SC technique ranges from 93 to 100% [24] which corresponds with the 100% survival rate observed in this study at the one-year follow-up. The results of our study are similar to those of Scipioni et al. [18], who published articles on edentulous ridge expansion with a 98.8% implant survival rate for over 5 years.

The mean age of our patients was 62.5 years. The results of our study showed that all the observed parameters in the study (ISQ, bone width, and bone density) were high and implant success rate was 100%. It may imply that age itself did not influence the bone healing or success rates of the dental implants.

There were no complications either during the operation or postoperatively. Previous studies reported fracture of the buccal cortical lamella as the most common intraoperative complication [25,26]. In our study, no fracture of the buccal bone lamella occurred, either in the mandible or in the maxilla. This may be explained by the use of an expansion technique that does not involve vertical osteotomies of the buccal cortical plate. In all cases, a longitudinal midcrestal osteotomy was performed, extending 1 cm beyond the planned implant bed. During expansion, this facilitated improved longitudinal stress distribution through the buccal bone lamella, thereby preventing fracture.

## 5. Conclusions

Considering the limitations of our preliminary study, we found that SC with simultaneous implantation was a successful and predictable technique either with or without filling the gap with bone substitutes, in situations with narrow alveolar ridges with sufficient height. While bone grafting may offer modest advantages for bone preservation, the use of dentin grafting did not demonstrate significant benefits and may lead to greater resorption. For better clinical relevance, further research with larger randomized samples, comparison with a variety of bone substitutes, and a longer follow-up period is needed.

## Figures and Tables

**Table 1:** Sample characteristics.

Patient	Age	Gender	Missing Teeth	Group
1	60	M	16, 17, 18	I
2	70	F	Edentulous maxilla	I
3	58	M	Edentulous mandible	III
4	61	F	34, 35, 36, 37, 38	II
5	59	M	Edentulous maxilla	III
6	68	M	Edentulous mandible	III
7	56	F	35, 36, 37, 38	II
8	55	F	35, 36, 37, 38	II
9	70	F	12, 11, 21, 22	I
10	68	M	Edentulous mandible	I
11	56	M	Edentulous mandible	III
12	69	M	Edentulous mandible	II
Mean	62.5			

**Table 2:** Differences between the groups in ISQ values, bone width, and bone density changes.

	ISQ (3 Months p.o vs. Immediate p.o) (Mean ± SD)	Bone Width (3 Months p.o vs. Immediate p.o (mm)) (Mean ± SD)	Bone Width (3 Months p.o vs. pre op (mm)) (Mean ± SD)	Bone Density (3 Months p.o vs. Immediate p.o (HU)) (Mean ± SD)	Bone Density (3 Months p.o vs. pre op (HU)) (Mean ± SD)
SC	+4.8 ± 6.2	−0.06 ± 0.08 ª	+2.46 ± 0.49	+154 ± 144	+459 ± 426
SC and bone graft	+4.0 ± 7.2	−0.49 ± 0.75	+2.18 ± 0.74	+89 ± 497	+388 ± 386
SC and dentin	−7.4 ± 5.2 * p < 0.05	−0.83 ± 0.69 *p* < 0.05	+1.52 ± 0.94	−41 ± 178	−198 ± 215 * p < 0.05

* Statistically significant difference to other groups; ª Statistically significant difference between the SC and SC and dentin groups.

## Data Availability

The data presented in this study are available on request from the corresponding author, due to privacy restrictions.
